# External Hemorrhage from a Portacaval Anastomosis in a Patient with Liver Cirrhosis

**DOI:** 10.1155/2014/523610

**Published:** 2014-07-08

**Authors:** Murat Biyik, Ramazan Ucar, Sami Cifci, Orhan Ozbek, Gokhan Gungor, Ozlem Ozer Cakir, Fatma Yavuz, Huseyin Ataseven, Ali Demir

**Affiliations:** ^1^Department of Gastroenterology, Meram School of Medicine, Necmettin Erbakan University, Meram, 42090 Konya, Turkey; ^2^Department of Immunology and Allergic Diseases, Meram School of Medicine, Necmettin Erbakan University, 42090 Konya, Turkey; ^3^Department of Radiology, Meram School of Medicine, Necmettin Erbakan University, 42090 Konya, Turkey; ^4^Division of Gastroenterology, Konya Education and Research Hospital, 42090 Konya, Turkey; ^5^Department of Internal Medicine, Meram School of Medicine, Necmettin Erbakan University, 42090 Konya, Turkey

## Abstract

Variceal bleeding is the major complication of portal hypertension in patients with liver cirrhosis. Hemorrhage mainly occurs in gastrointestinal lumen. Extraluminal hemorrhages are quite rare, such as intraperitoneal hemorrhages. We aimed to present a variceal bleeding case from the anastomosis on the anterior abdominal wall, as an extraordinary bleeding location, in a patient with portal hypertension in whom there were no esophageal and gastric varices.

## 1. Introduction

Among the complications of chronic liver disease due to portal hypertension are variceal bleeding, ascites, and hepatic encephalopathy. Variceal bleeding occurs mainly from the esophageal and gastric veins, but in rare cases bleeding into the intraperitoneal region may occur. We describe here a patient with external bleeding in the anterior abdominal wall arising from the anastomosis between the splenic and epigastric veins.

## 2. Case

**Figure 1:**
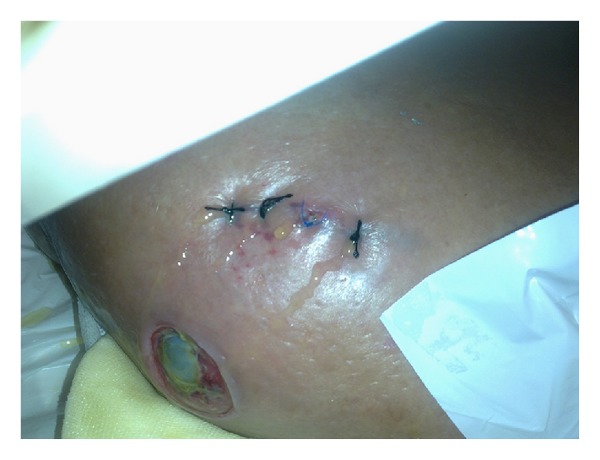
External hemorrhage from periumbilical anastomosis on the anterior abdominal wall.

**Figure 2:**
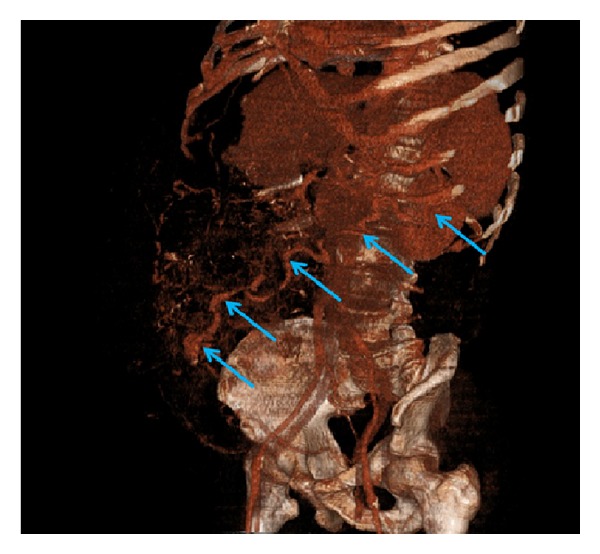
Aberrant venous collaterals between umbilical and splenic veins are shown with arrows.

A 61-year-old man with an 8-year history of cryptogenic liver cirrhosis was admitted to the emergency department with massive hemorrhage from the anterior abdominal wall. Physical examination showed blood pressure 80/50 mm Hg, heart rate 96/min, massive ascites, and bleeding from a vessel around the umbilicus (Figure 1). Laboratory parameters included hemoglobin 10.9 g/dL, platelets 121000 *μ*u/L, INR 1.6, albumin 2.3 g/dL, total bilirubin 2.9 mg/dL, AST 44 *μ*u/L, and ALT 14 *μ*u/L. The bleeding vessel was sutured by a cardiovascular surgeon and hemorrhage control was achieved. Subsequent medical treatment included infusions of somatostatin and human serum albumin and transfusions with erythrocyte suspensions and fresh frozen plasma. Gastroscopy performed after the patient stabilized revealed portal hypertensive gastropathy but no esophageal varices. Abdominal computerized tomography showed collaterals in the umbilical region originating from the anastomosis between the splenic and epigastric veins (Figure 2). The patient was discharged. One month later, however, he was admitted to the emergency department with hepatic encephalopathy and died.

## 3. Discussion

Portal hypertension is defined as a pressure above 12 mm Hg in the portal vein, leading to portosystemic shunts in several anatomic regions. The most common sites are between the gastroesophageal vein and azygos/hemiazygos system, between the hemorrhoidal and internal iliac veins, and between the umbilical and periumbilical veins draining into the epigastric veins of the anterior abdominal wall [1, 2].

Bleeding due to portosystemic shunts mainly results from esophageal and gastric varices. In rare cases, intraperitoneal and retroperitoneal hemorrhage due to portal hypertension have been reported [3]. The first case of intra-abdominal hemorrhage resulting from cirrhosis was reported in 1958 [4]. While esophageal variceal bleeding mainly presents with hematemesis and melena, hemoperitoneum presents with abdominal pain and distention, hypotension, and hemorrhagic shock [5]. The diagnosis of hemoperitoneum is established by paracentesis, Doppler ultrasonography, and computed tomography [6]. Because of the limited number of cases to date, optimum therapy has not been determined. Most patients with hemoperitoneum have been treated surgically [1]. Hepatic functional reserve, the occurrence of hemorrhagic shock, and early surgical intervention are important prognostic factors in patients with hemoperitoneum [5].

Bleeding in our patient occurred from the collaterals between the splenic and epigastric veins on the anterior wall of the abdomen, an area in which bleeding has not, to our knowledge, been reported in a patient with portal hypertension. This patient is therefore the first with liver cirrhosis to experience external hemorrhage from a portacaval anastomosis. Initial treatment in such patients should be based on achieving hemostasis by surgical intervention on the bleeding vessel, hemodynamic stabilization with erythrocyte suspensions and fluid replacement, and correction of coagulopathy with fresh frozen plasma. Portal hypertension may be reduced by vasoconstrictors such as somatostatin and terlipressin [7].
